# Comprehensive spatial and immune profiling of metastatic mismatch repair–deficient colorectal cancer reveals response to immunotherapy

**DOI:** 10.1093/immadv/ltag001

**Published:** 2026-01-09

**Authors:** Ilkyu Park, Hari Kang, Se-Hee Kim, YunJae Jung, Won-Suk Lee

**Affiliations:** Department of Medicine, College of Medicine, Gachon University, Incheon 21565, Republic of Korea; Department of Surgery, Gachon University Gil Medical Center, Incheon 21565, Republic of Korea; Gachon Medical Research Institute, Gachon University Gil Medical Center, Incheon 21565, Republic of Korea; Department of Microbiology, College of Medicine, Gachon University, Department of Health Science and Technology, Gachon Advanced Institute for Health Science & Technology, Incheon, Republic of Korea; Department of Surgery, Gil Medical Center, Gachon University College of Medicine, Incheon, Republic of Korea

**Keywords:** mismatch repair deficiency, colorectal cancers, immune checkpoint inhibitors, multiplexed immunohistochemistry, spatial immune profiling

## Abstract

**Introduction:**

Mismatch repair-deficient (dMMR) colorectal cancers (CRCs) exhibit variable clinical responses to immune checkpoint inhibitors (ICIs) despite their high immunogenicity.

**Methods:**

To investigate the molecular and spatial determinants of this heterogeneity, we analyzed five metastatic sites (colon, liver, peritoneum, left ovary, and right ovary) from a dMMR CRC patient treated with pembrolizumab using bulk and single-cell RNA sequencing combined with multiplex immunohistochemistry.

**Results:**

Responsive lesions were characterized by enriched cytotoxic and interferon-γ signatures, greater CD8^+^ T-cell infiltration, and close spatial proximity between programmed death-ligand 1(PD-L1)^+^ tumor cells and M1-like macrophages. In contrast, resistant lesions demonstrated reduced effector cell presence and were enriched in immunosuppressive programs, including SPP1^+^ and CD163^+^ macrophages, noncanonical WNT5A/B signaling, and TGF-β–mediated matrix remodeling.

**Conclusion:**

Collectively, these findings highlight the importance of spatial immune architecture and macrophage polarization in shaping ICI responses in dMMR CRC and underscore the need for spatial profiling to guide immunotherapy strategies in metastatic disease.

## Graphical Abstract

**Graphical Abstract:**
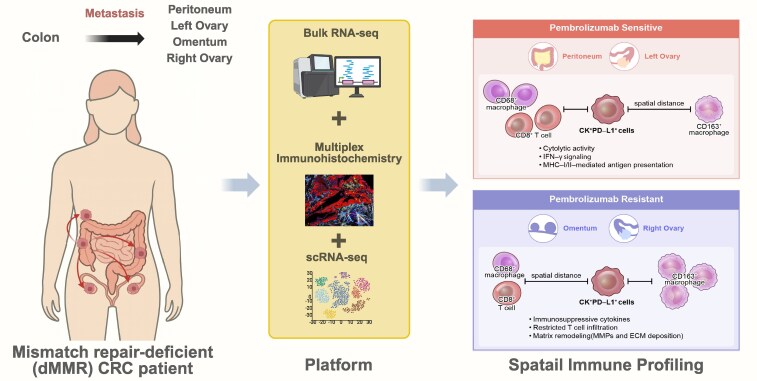

## Introduction

Colorectal cancer (CRC) is a genetically and immunologically heterogeneous disease, with mismatch repair deficiency (dMMR) representing 4%–5% of metastatic cases [1, 2]. dMMR arises from the inactivation of key DNA repair genes such as MLH1, PMS2, MSH2, and MSH6, resulting in microsatellite instability-high (MSI-H) and an elevated tumor mutational burden (TMB) [3, 4]. These tumors typically generate abundant neoantigens and show high infiltration of CD8^+^ T cells and PD-L1 expression, often conferring immune responsiveness [5]. Despite this, metastatic spread can occur, and clinical responses to immune checkpoint inhibitors (ICIs) remain variable.

ICIs targeting the PD-1/PD-L1 axis, such as pembrolizumab and nivolumab, have shown significant clinical efficacy in dMMR CRC, gaining FDA approval for first- and second-line treatment [6, 7]. Combination regimens, including neoadjuvant nivolumab plus ipilimumab, have demonstrated remarkable response rates (∼95%) [8]. However, objective response rates remain suboptimal (31%–33%), highlighting the need to better understand the mechanisms of ICI resistance [9, 10].

Tumor-associated macrophages (TAMs) are key components of the tumor microenvironment (TME) that modulate ICI responsiveness. Recruited via cytokines such as CCL2, CXCL12, VEGF, and TGF-β, TAMs exhibit plasticity and polarize into either pro-inflammatory M1 or immunosuppressive M2 phenotypes depending on environmental cues [11, 12]. In CRC, TAMs predominantly adopt an M2 phenotype, supporting immune suppression, tumor progression, and metastasis. M2-polarized TAMs recruit regulatory T cells (Tregs), inhibit CD8^+^ T-cell and NK-cell function, and express checkpoint ligands like PD-L1 and enzymes such as ARG1 and NOS, which impair cytotoxic activity [13, 14]. High densities of M2 TAMs are associated with poor prognosis and limited ICI response in both early- and advanced-stage CRC [15].

To investigate the mechanisms of responsiveness and resistance to ICIs, we performed spatial proteomic and single-cell transcriptomic analyses of multiple metastatic lesions from a patient with dMMR CRC who exhibited heterogeneous responses to pembrolizumab. Our study aimed to dissect the immune context and functional architecture of TAMs in shaping local ICI efficacy, providing insight into therapeutic strategies for overcoming resistance in metastatic dMMR CRC.

## Materials and methods

### Clinical information

A 43-year-old woman was diagnosed with an obstructing ascending colon cancer with resectable liver metastasis. This study encompasses her clinical course from the initial diagnosis in March 2018 through curative resection, palliative chemotherapy, and immunotherapy with pembrolizumab administered between December 2020 and May 2021, accompanied by interval CT evaluations. She subsequently remained under active surveillance, with regular imaging and clinical follow-up extending through May 2025. Following the initial resection, the patient received 12 cycles of palliative chemotherapy with FOLFOX. Two years later, the tumor recurred at multiple sites, including the ovaries, the peritoneum, and the omentum. She received four cycles of pembrolizumab and exhibited heterogeneous responses across metastatic sites—regression in the peritoneum and left ovary, but resistance in the omentum and right ovary. All metastatic lesions evaluated in this study were obtained during a single interval debulking surgery performed after the patient had completed four cycles of pembrolizumab. The right ovary, left ovary, omentum, and peritoneal lesions were resected concurrently, indicating that all specimens represent synchronous metastatic sites collected at an identical therapeutic timepoint. Five years after the recurrence, the patient remains disease-free, with no evidence of progression or relapse, suggesting durable complete remission. dMMR status was confirmed by immunohistochemistry for MLH1, PMS2, MSH2, and MSH6. This study was approved by the Institutional Review Board of Gachon University Gil Medical Center (approval number: GCIRB2024-021). All procedures were conducted in accordance with the ethical standards of the institutional research committee, the Good Clinical Practice Guidelines, and the Declaration of Helsinki. Written informed consent was obtained from the patient prior to participation in the study.

### Tumor sample collection and RNA sequencing

Tumor specimens from surgical resections or biopsies with ≥20% tumor content were processed for RNA extraction (QIAamp Mini Kit). RNA-seq libraries were prepared using Illumina TruSeq kits and sequenced on the HiSeq 2500 platform (2 × 50 bp). Reads were aligned to the GRCh38 genome using STAR (v2.6.1) and quantified as TPM using RSEM (v1.3.1), following GTEx-recommended parameters.

### Immunohistochemistry for mismatch repair-deficient and PD-L1

FFPE tissues were sectioned (4 μm), mounted on Superfrost Plus slides, and dried at 60°C. IHC for MLH1, PMS2, MSH2, and MSH6 was performed as previously reported [16], with loss of one or more proteins indicating dMMR. PD-L1 IHC was conducted on a Dako Autostainer Link 48 using the PD-L1 IHC 22C3 pharmDx Kit and EnVision FLEX detection. Combined positive score (CPS) was calculated for tumor and immune cells; CPS ≥1 was considered PD-L1-positive cells using IHC. The specimen was considered PD-L1-positive when CPS was ≥ 1.

### Whole transcriptome sequencing and analysis

RNA libraries were prepared using the TruSeq Stranded Total RNA kit with Ribo-Zero Gold and sequenced on Illumina HiSeq 2500 (2 × 50 bp). Libraries were normalized to 20 pM and sequenced using V3 chemistry. Reads were aligned to the GRCh38 human genome with STAR (v2.6.1) [17] and quantified as TPM using RSEM (v1.3.1) [18], following GTEx guideline

### Multiplex immunofluorescence and image analysis

FFPE sections were stained using the Opal Polaris 7-color kit (Akoya) on a Leica BOND Rx autostainer. Primary antibodies included panCK/Opal-620, PD-L1/Opal-480, CD8/Opal-780, CD68/Opal-690, CD11C/Opal-520, and CD163/Opal-570. Slides were counterstained with DAPI and imaged with spectral unmixing. Image analysis was performed using inForm software (PerkinElmer), with tissue segmentation into CK^+^ tumor and CK⁻ stromal regions. Cell segmentation was performed based on nuclear boundaries followed by phenotype assignment using a supervised classifier trained on representative tumor and stromal regions to minimize arbitrary intensity cut-offs. Spectral unmixing, background subtraction, and batch correction were conducted using optimized default parameters in inForm. Cells were not positive for any of the six markers designated as unassigned and are reported. Spatial proximity metrics were quantified using the phenoptr R package (v0.3.2). Centroid-to-centroid nearest-neighbor distances were calculated between CK^+^PD-L1^+^ tumor cells and immune subsets, and the number of neighboring cells within a fixed 15-µm radius was summarized to assess local immune–tumor interactions. Representative segmentation outputs and nearest-neighbor maps are also provided.

### Single-cell RNA sequencing and analysis

Tumor tissues were dissociated using the gentleMACS system and cryopreserved. Single-cell libraries were prepared using the 10 × Genomics Chromium 5′ V(D)J platform and sequenced on NovaSeq 6000 (∼50 000 reads/cell). Reads were mapped to GRCh38 with CellRanger (v5.0). Analysis was conducted in R (v4.1) using Seurat (v4.0) [19]. Low-quality cells (<300 genes or >40% mitochondrial content) were excluded. Normalization, scaling, and clustering were performed with batch correction using Harmony [20]. Cell types were annotated based on canonical markers using the FindAllMarkers function.

### Cell–cell communication analysis

Cell–cell communication was inferred using the CellChat R package (v2.1.2). Normalized scRNA-seq data from each metastatic lesion were used to construct independent CellChat objects, and ligand–receptor interactions were analyzed using the built-in human secreted signaling, ECM, and cell–cell contact databases. For each dataset, overexpressed ligands, receptors, and interaction pairs were identified, and communication probabilities were estimated using CellChat’s permutation-based statistical framework. Significant interactions (*P* < 0.05) were retained for downstream analysis, and pathway-level signaling was inferred by aggregating ligand–receptor pairs into curated signaling modules. Communication strength, information flow, and sender/receiver roles were computed using the default pipeline. For comparing pembrolizumab-resistant and -sensitive lesions, communication probabilities were averaged within each group, and differential pathway activity was summarized using log₂ fold-change and CellChat’s built-in comparison functions.

### Statistical analysis

Statistical analyses were performed in R (v4.1.1). Continuous variables were compared using the Wilcoxon signed-rank test or one-way ANOVA, as appropriate. Trend analyses were conducted using linear regression-based trend tests (and ANOVA-based trend assessment where applicable). Two-sided *P*-values < .05 were considered statistically significant.

## Results

### Heterogeneous pembrolizumab response across metastatic sites in mismatch repair-deficient colorectal cancer

**Figure 1:**
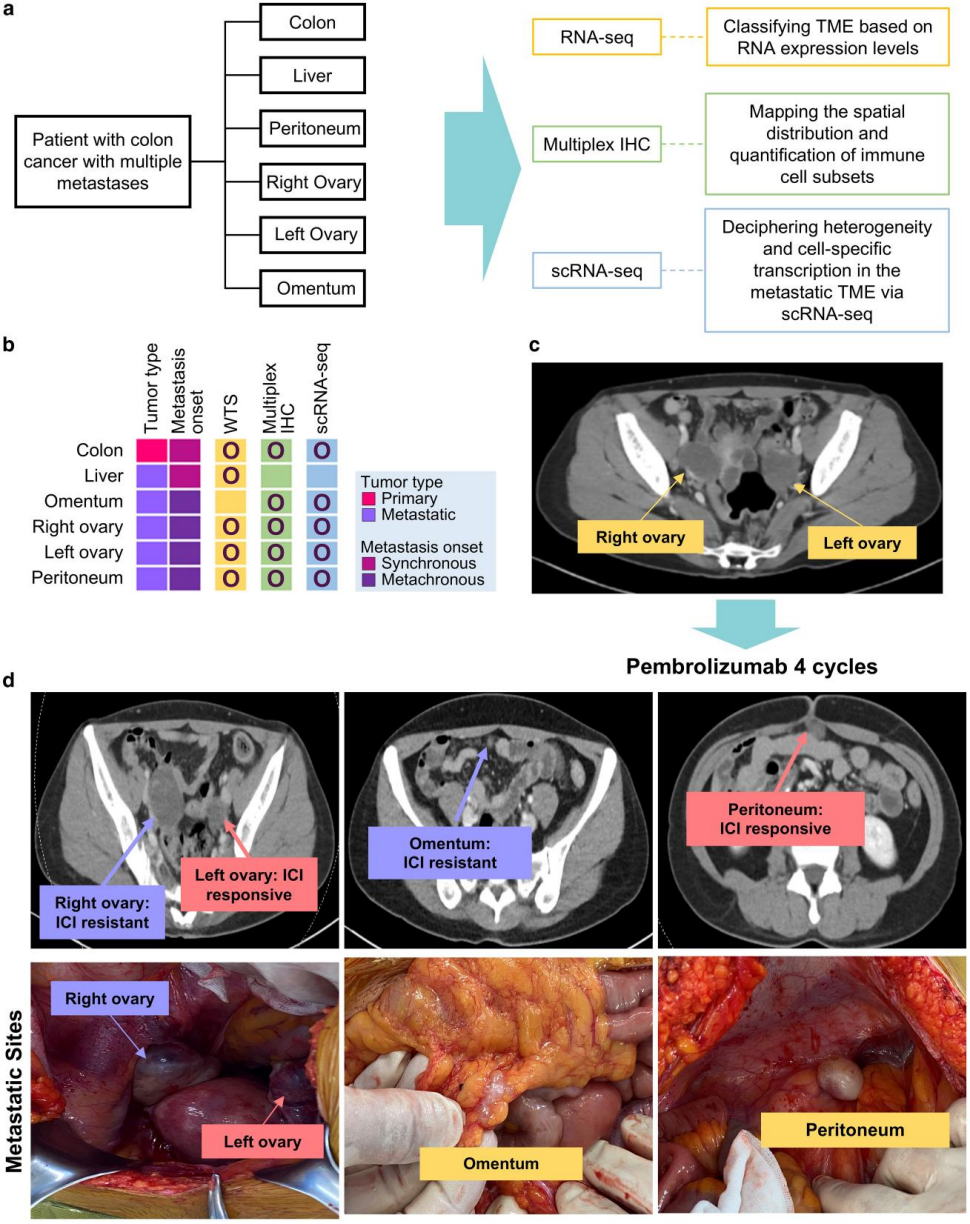
Comprehensive multi-omic analysis and imaging of metastatic dMMR CRC. Metastatic CRC with dMMR, involving multiple anatomical sites including the liver, peritoneum, ovary, and omentum, was characterized using an integrative multi-omic and spatial profiling approach. (a) Study overview depicting the integrated multi-omic design and corresponding tissue sampling sites. (b) Schematic representation of metastatic sites with associated sample types and analytical platforms. (c) Preoperative CT imaging showing bilateral ovarian metastases prior to immune checkpoint blockade. (d) Postoperative evaluation of metastatic lesions following four cycles of pembrolizumab, including representative tissue image (left) and matched CT scan (right). Labels such as left ovary and right ovary indicate anatomical origin of lesions and are not related to the vertical placement of images.

To investigate the organ-specific heterogeneity of response to ICIs in dMMR CRC, we conducted a comprehensive spatial and transcriptomic analysis across multiple metastatic sites exhibiting variable therapeutic responses to pembrolizumab. Using tissue specimens resected from the colon, liver, omentum, peritoneum, and bilateral ovaries, we applied an integrative multi-modal strategy comprising bulk RNA sequencing, single-cell RNA sequencing, and multiplex immunohistochemistry (Fig. 1a). Each lesion was profiled in parallel to preserve spatial resolution and enable direct comparison of molecular and cellular features across metastatic niches (Fig. 1b).

We applied this integrative spatial and transcriptomic approach to a 43-year-old female patient with dMMR CRC who initially received FOLFOX chemotherapy for liver-limited disease but developed multifocal relapse 2 years later. Pretreatment imaging revealed bilateral ovarian metastases (Fig. 1c). Following four cycles of pembrolizumab, site-specific responses were observed—tumor regression in the peritoneum and left ovary, but radiographic persistence in the omentum and right ovary (Fig. 1d). Salvage surgery enabled systematic evaluation of both synchronous and metachronous lesions within a single immune landscape.

### Immune state and response-associated transcriptional programs distinguish metastatic lesions in mismatch repair-deficient colorectal cancer

**Figure 2:**
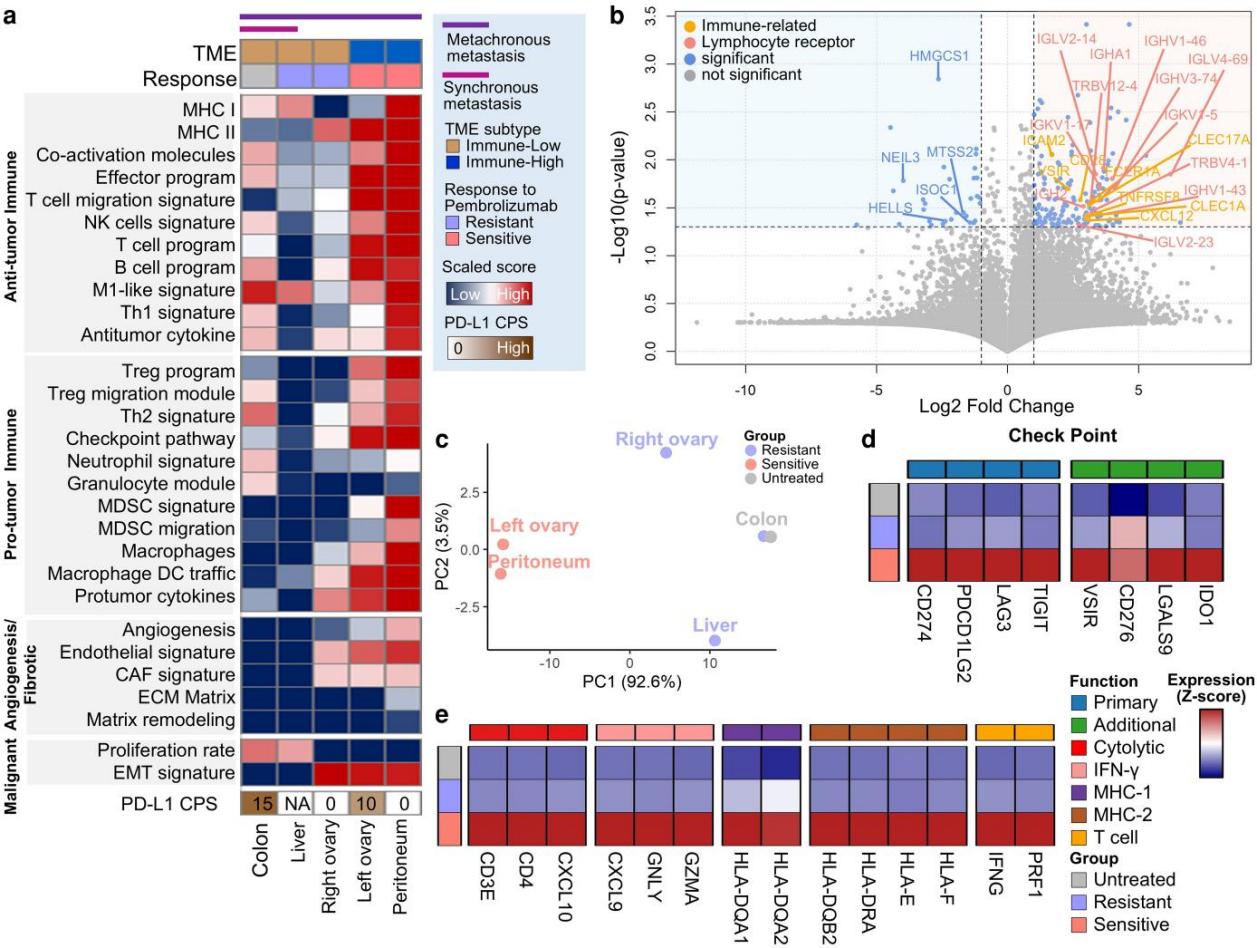
Functional characterization of immune heterogeneity and checkpoint blockade responsiveness in metastatic dMMR CRC. (a) Functional transcriptomic profiling of metastatic tumor samples stratified by immune infiltration status (immune high vs. immune low). (b) Volcano plots highlighting differentially expressed genes between immune-high and immune-low tumors. (c) PCA reveals distinct clustering of samples based on pembrolizumab sensitivity, distinguishing sensitive from resistant phenotypes. (d, e) Expression patterns of key immune-associated genes were evaluated across distinct treatment response groups (f) Multiplex immunohistochemistry (mIHC) analysis of metastatic lesions using epithelial (CK) and immune markers (PD-L1, CD8, CD68, CD163, and CD11c), displaying integrated tissue maps (far left) and marker-specific distributions for each region.

To investigate the molecular correlates of variable responses to pembrolizumab across metastatic lesions, we performed transcriptomic profiling of five tumor sites: colon, liver, right ovary, left ovary, and peritoneum (Fig. 2a). Pembrolizumab-sensitive lesions (peritoneum and left ovary) displayed broad enrichment of anti-tumor-immune gene signatures, including transcriptional modules associated with effector T and NK cell activity, T-cell migration signatures, B cell-associated programs, and M1-like macrophage signatures. Notably, immunoregulatory gene modules—such as those associated with Treg programs, MDSC-associated modules, Th2 polarization, and immune checkpoint pathways—were also elevated in the same tumors, suggesting an inflamed but heterogeneous immune context rather than a uniformly polarized state. In addition to immune-associated modules, stromal remodeling programs—including angiogenesis, endothelial signatures, and fibroblast-associated programs—were elevated in responsive lesions, with partial enrichment observed in the right ovary. Proliferation-associated modules were most prominent in the colon and liver, whereas EMT-related signatures were elevated across all pelvic lesions, irrespective of clinical response.

To further dissect these patterns, samples were stratified into immune-high and immune-low categories based on aggregated immune signature scores (Fig. 2b). Genes upregulated in immune-high tumors—primarily derived from pembrolizumab-responsive lesions—were enriched for lymphocyte migration and activation programs (e.g. CXCL12, CD28, and TNFRSF8), as well as immunoglobulin and T-cell receptor components (e.g. IGHA1, IGHV1-46, and TRBV4-1), and additional immune regulatory molecules (e.g. VSIR, ICAM2, and CLEC1A). Conversely, immune-low lesions showed increased expression of metabolic and chromatin-regulating genes (e.g. HELLS, HMGCS1, and NEIL3), consistent with a less immune-infiltrated transcriptional state.

To assess whether global transcriptional profiles correspond to treatment response, we performed principal component analysis (PCA) across the five metastatic lesions (Fig. 2c). Tumors from the left ovary and peritoneum—both responsive to pembrolizumab—clustered closely together and were distinct from immune-low, non-responsive tumors such as the right ovary, liver, and colon, suggesting shared transcriptional programs associated with sensitivity.

According to pembrolizumab response, we next compared expression levels of immune checkpoint, cytolytic, interferon, and antigen-presentation-related genes across untreated, resistant, and sensitive tumors (Fig. 2d-e). Canonical checkpoint molecules (CD274, PDCD1LG2, LAG3, and TIGIT) and additional regulators (VSIR, CD276, LGALS9, and IDO1) were consistently upregulated in the sensitive group. Similarly, cytolytic effectors (GZMA, PRF1, and GNLY), IFN-γ response genes (CXCL9, CXCL10, and IFNG), MHC class I and II molecules, and T-cell-associated transcripts (CD3D and CD8A) were all elevated in sensitive tumors but minimally expressed in resistant lesions and the untreated primary.

To assess whether this transcriptional activation corresponds to actual immune infiltration, we performed cell-type deconvolution of bulk RNA-seq using EPIC (Supplementary Figure S2). Colon and liver lesions were largely composed of uncharacterized (non-immune) transcriptomes with minimal inferred immune or stromal fractions. In contrast, pembrolizumab-responsive lesions (peritoneum and left ovary) showed the highest estimated proportions of CD8^+^/CD4^+^ T cells, macrophages, endothelial cells, and cancer-associated fibroblasts, whereas the right ovary exhibited an intermediate profile. Together, these analyses indicate that sensitive lesions are not only transcriptionally immune-activated but also globally more immune-infiltrated, whereas resistant sites remain comparatively immune-poor despite their shared clonal origin.

### Spatial architecture of immune–tumor interfaces differs by pembrolizumab responsiveness

**Figure 3:**
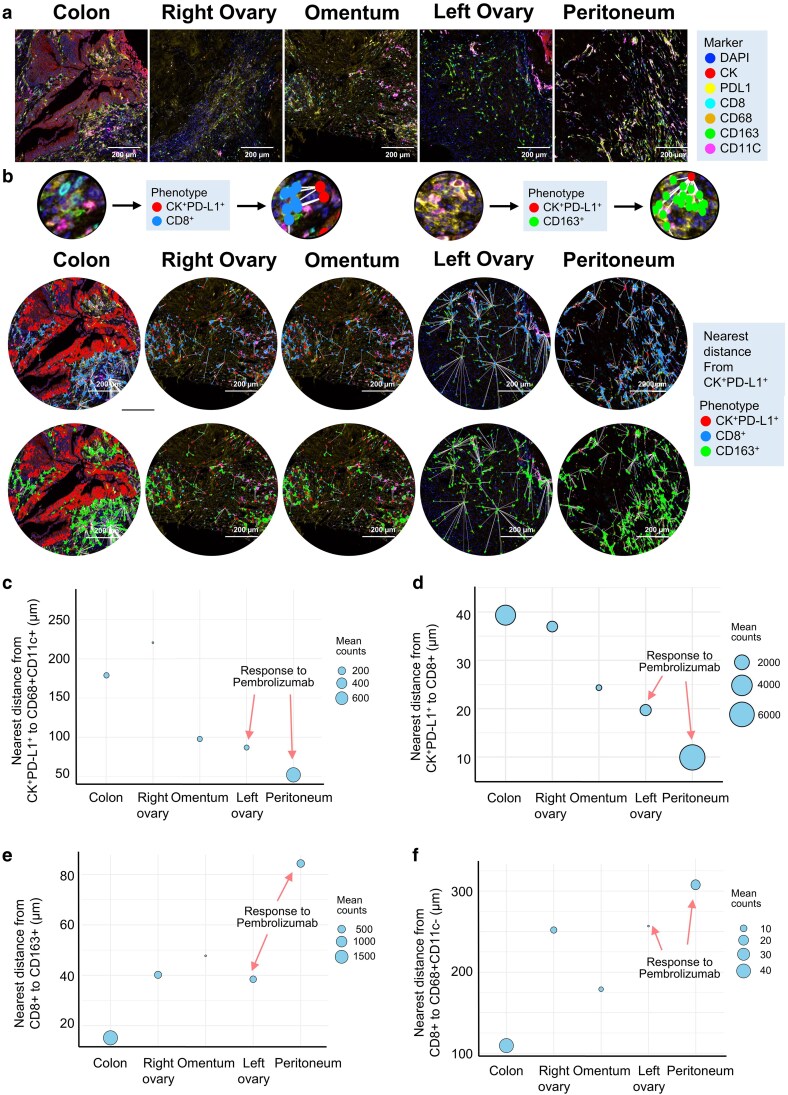
Spatial proximity analysis of PD-L1^+^ tumor cells and immune subsets in metastatic dMMR CRC. (a) Representative multiplex immunohistochemistry (mIHC) images depicting spatial localization of PD-L1^+^ epithelial tumor cells (CK^+^PD-L1^+^), cytotoxic T cells (CD8^+^), and M2-like macrophages (CD163^+^) across metastatic regions. DAPI, CK, PD-L1, CD8, CD68, CD163, and CD11c markers were used for segmentation and phenotyping. Nearest distances from CK^+^PD-L1^+^ tumor cells to CD8^+^ and CD163^+^ immune cells are visualized with white connecting lines. (b–e) Quantitative spatial analyses measuring the nearest distances (in µm) between immune and tumor compartments across distinct metastatic sites. (b) Distance from CK^+^PD-L1^+^ cells to CD68^+^CD11c^+^ myeloid cells. (c) Distance from CK^+^PD-L1^+^ cells to CD68^+^CD11^+^ cells. (d) Distance from CK^+^PD-L1^+^ cells to CD8^+^ T cells. (e) Distance from CD8^+^ T cells to CD163^+^ macrophages. (f) Distance from CD8^+^ T cells to CD68^+^CD11c⁻ macrophages.

To investigate spatial determinants of immune responsiveness in dMMR CRC, we performed multiplex immunohistochemistry (mIHC) on metastatic lesions from five anatomical sites (colon, right ovary, omentum, left ovary, and peritoneum). Marker panels included cytokeratin (CK), PD-L1, CD8, CD68, CD163, and CD11c, enabling phenotypic identification of epithelial tumor cells (CK^+^PD-L1^+^), cytotoxic T cells (CD8^+^), and macrophage subsets including M1-like (CD68^+^CD11c^+^) and M2-like (CD163^+^) populations (Fig. 3a; Supplementary Figure S3-1 to S3-3).

We quantified immune infiltration using mIHC-derived cell densities per tumor area Supplementary Figure S4). Pembrolizumab-responsive lesions (peritoneum and left ovary) exhibited the highest densities of CD8^+^ T cells and M1-like macrophages, whereas resistant lesions (omentum and right ovary) showed abundant CK⁻/PD-L1^+^ stromal cells but lower effector density. The primary colon lesion was tumor-dense but immune-poor. These density measurements are broadly consistent with the trends observed in bulk RNA-seq deconvolution and offer a complementary, tissue-level view of lesion-specific immune infiltration.

Quantitative spatial proximity analysis was then conducted to assess immune–tumor architecture across metastatic lesions (Fig. 3b–f). White connecting lines demarcated the nearest spatial distances between CK^+^PD-L1^+^ tumor cells and neighboring immune populations, revealing site-specific differences in immune infiltration. Pembrolizumab-sensitive lesions—particularly the peritoneum and left ovary—exhibited significantly reduced distances between CK^+^PD-L1^+^ tumor cells and CD68^+^CD11c^+^ M1-like macrophages (Fig. 3c) as well as between tumor cells and CD8^+^ T cells (Fig. 3d). These findings suggest enhanced recruitment and colocalization of effector immune cells within the tumor microenvironment in responsive regions.

In contrast, analysis of immune–stromal relationships revealed distinct patterns of spatial exclusion. In pembrolizumab-sensitive sites, CD8^+^ T cells were located further away from CD163^+^ M2-like macrophages (Fig. 3e) and CD68^+^CD11c⁻ macrophages (Fig. 3f), indicating reduced exposure to immunosuppressive myeloid populations within the stromal compartment. Conversely, in resistant lesions such as the colon and right ovary, these distances were shorter, suggesting potential inhibitory crosstalk. These spatial configurations collectively suggest that effective anti-tumor immunity in responsive metastatic sites may be driven by both enhanced effector cell–tumor interactions and spatial separation from suppressive stromal macrophages.

### Single-cell transcriptomic dissection reveals differential immune composition and macrophage polarization associated with pembrolizumab response in metastatic mismatch repair-deficient colorectal cancer

**Figure 4:**
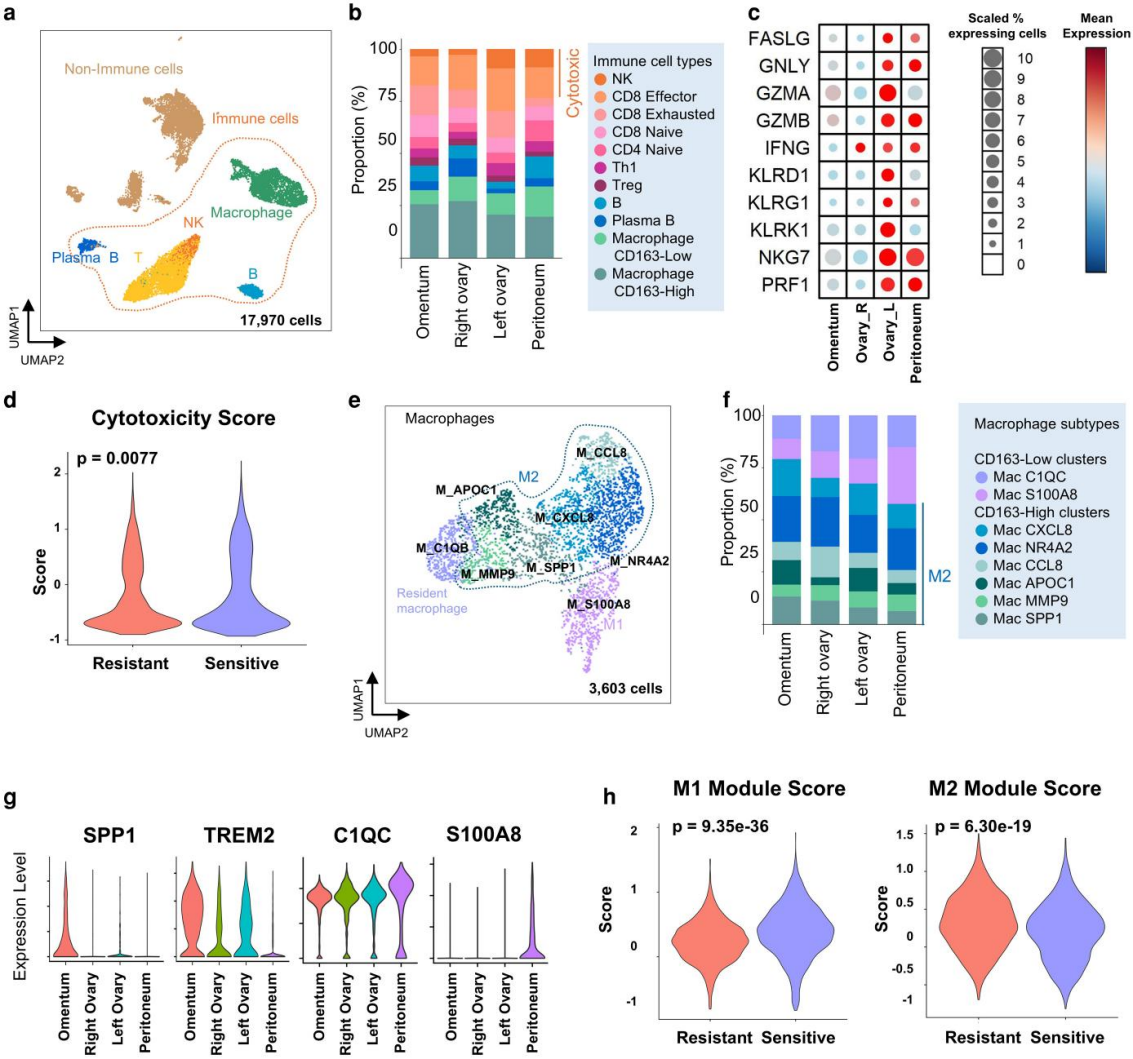
Single-cell transcriptomic dissection of immune composition, cytotoxicity, and macrophage polarization in metastatic dMMR CRC. (a) UMAP projection of single-cell transcriptomes showing the distribution of immune and non-immune cells across metastatic tissues. Immune cells are distinguished from epithelial and stromal compartments. (b) Relative proportions of annotated immune cell types. (c) Expression of representative cytotoxicity-associated genes in T and NK cells across metastatic sites. (d) Cytotoxicity scores compared between pembrolizumab-sensitive and -resistant groups. (e) UMAP embedding of macrophages highlighting distinct subpopulations defined by canonical marker genes. (f) Distribution of macrophage subpopulations across different metastatic sites. (g) Expression of M1- and M2-associated genes across tissues. (h) Module scores representing M1 and M2 polarization states, stratified by ICI response status.

To comprehensively characterize the immune landscape underpinning variable responses to pembrolizumab, we performed single-cell RNA sequencing (scRNA-seq) on immune-enriched populations from four metastatic lesions (right ovary, left ovary, omentum, and peritoneum). A total of 17 970 high-quality single-cell transcriptomes were obtained, and unsupervised clustering delineated seven major cellular compartments—T cells, NK cells, macrophages, B cells, plasma cells, epithelial cells, and stromal cells—based on canonical lineage markers (Fig. 4a; Supplementary Figure S5). Among immune subsets, pembrolizumab-sensitive sites (left ovary and peritoneum) exhibited markedly elevated proportions of NK cells compared to resistant lesions (right ovary and omentum) (Fig. 4b), suggesting a link between NK cell abundance and therapeutic responsiveness. These single-cell compositional differences were concordant with both EPIC-based deconvolution of bulk RNA-seq and mIHC-derived cell density measurements, which similarly indicated increased immune infiltration in pembrolizumab-responsive lesions (Supplementary Figures S2 and S4).

We next assessed cytotoxicity-related transcripts in T and NK cells across metastatic sites (Fig. 4c). Cytotoxic effector molecules, including GZMB, PRF1, GNLY, IFNG, and CD8A/B, were upregulated in immune cells from pembrolizumab-sensitive lesions, indicative of a more active cytolytic program. Consistently, the composite cytotoxicity score was higher in sensitive lesions compared to resistant ones (*P* = .007; Fig. 4d).

To investigate the role of macrophage polarization in modulating pembrolizumab response, we focused our analysis on macrophage populations (Fig. 4e). Notably, multiple M2-like subclusters (Supplementary Figure S4) were markedly reduced in pembrolizumab-sensitive lesions such as the left ovary and peritoneum (Fig. 4f), suggesting a shift away from immunosuppressive phenotypes in responsive sites. We next evaluated gene expression patterns of key M1- and M2-associated transcripts across sites (Fig. 4g). SPP1 and TREM2, representing M2-like polarization, were most prominently expressed in omental lesions, whereas M1-related markers such as C1QC and S100A8 were highly enriched in sensitive regions, including the peritoneum and left ovary. Finally, polarization scores calculated using M1 and M2 gene signatures revealed reciprocal trends: M1 module scores were significantly elevated in sensitive tumors (*P* = 9.35e-36), while M2 scores were higher in resistant lesions (*P* = 6.30e-19) (Fig. 4h). These findings demonstrate that pembrolizumab-sensitive metastases are enriched for cytotoxic lymphocytes and M1-like macrophages, whereas resistant tumors harbor immunosuppressive macrophage states and diminished effector function.

### Intercellular signaling networks reveal immune-suppressive versus immune-activating states in immune checkpoint inhibitor-resistant and -responsive tumors

**Figure 5:**
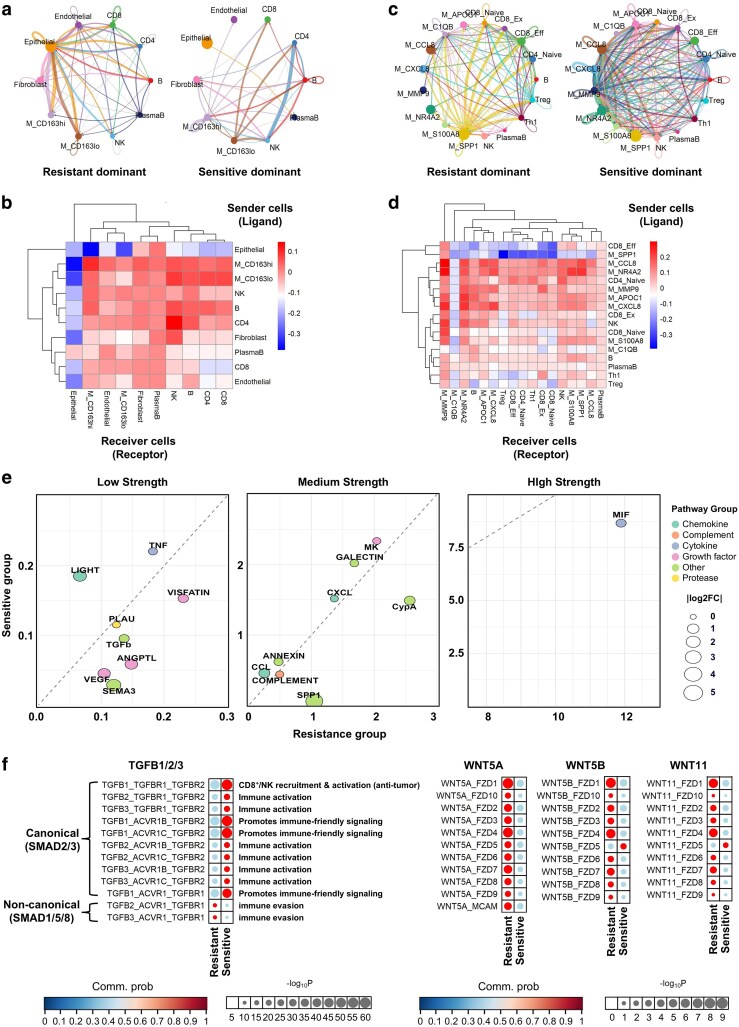
Intercellular communication landscapes distinguish ICI-responsive and resistant tumor ecosystems. (a) Circle plots showing global cell–cell interaction networks preferentially dominant in pembrolizumab-resistant (left) and pembrolizumab-sensitive (right) tumors. Edge thickness corresponds to the strength of predicted ligand–receptor interactions among major cell populations. (b) Heatmap illustrating differential interaction strength between sender (ligand-expressing) and receiver (receptor-expressing) cell types, with red intensity indicating greater signaling activity in the sensitive group relative to the resistant group. (c) Immune cell-restricted circle plots visualizing intercellular communication among annotated immune subpopulations, separately for resistant-dominant (left) and sensitive-dominant (right) networks. Line width reflects interaction strength. (d) Heatmap showing differential signaling activity across immune subpopulations, arranged by sender (*y*-axis) and receiver (*x*-axis), highlighting signals preferentially active in sensitive tumors. (e) Bubble plot summarizing the relative strength of signaling pathways across resistant (*x*-axis) and sensitive (*y*-axis) groups, categorized by overall intensity (low, medium, and high). Bubble size represents the log₂ fold-change in communication strength. (f) Paracrine signaling pathways across resistant and sensitive tumors. Selected axes (TGFB1/2/3; WNT5A, WNT5B, and WNT11) are compared with ligand–receptor pairs on the *y*-axis and response groups on the x-axis.

To elucidate how tumor-immune crosstalk contributes to differential responses to pembrolizumab, we performed cell–cell communication analysis using single-cell transcriptomic data from four metastatic lesions. Global intercellular interaction networks revealed striking differences between pembrolizumab-resistant and -sensitive tumor ecosystems (Fig. 5a, Supplementary Figure S5). In resistant tumors, epithelial cells exhibited dense interactions with diverse stromal and immune compartments, suggesting a tumor-dominant signaling architecture. In contrast, sensitive tumors were characterized by more extensive immune–immune communication, particularly involving CD4^+^ T cells, NK cells, and macrophages, indicative of a more immunologically coordinated microenvironment.

Quantitative comparison of interaction strength between sender and receiver cell types further highlighted these distinctions (Fig. 5b). In sensitive tumors, most immune cell populations—such as CD8^+^ T cells, B cells, and macrophages—exhibited stronger outgoing and incoming signals relative to resistant sites. Epithelial cells, by contrast, showed limited signaling activity in the sensitive group, with the exception of interactions with plasma B cells. These findings underscore a shift in signaling dominance from tumor epithelium to immune constituents in ICI-responsive niches.

When restricted to immune populations (Fig. 5c), pembrolizumab-sensitive tumors exhibited enhanced intercellular communication across multiple immune subsets, including CD4^+^ T cells, NK cells, and non-SPP1^+^ macrophage subtypes. In contrast, SPP1^+^ TAMs dominated the signaling landscape in resistant tumors, engaging in broad interactions with various immune populations. Notably, in sensitive tumors, SPP1^+^ TAMs were relatively isolated from other immune compartments, suggesting reduced immunosuppressive influence. Similarly, C1QB^+^ macrophages showed attenuated connectivity in sensitive lesions, consistent with a microenvironment characterized by diminished suppressive macrophage crosstalk and increased coordination among effector immune cells.

Sender–receiver mapping at the immune subtype level revealed selectively enhanced interactions in the sensitive group (Fig. 5d). For example, communication from MMP9^+^ macrophages to CCL8^+^ macrophages, and sequentially from CCL8^+^ macrophages to APOC1^+^ subsets reflected a strengthened monocyte–monocyte axis. Additionally, naïve CD4^+^ T cells in the sensitive group actively signaled to CD8^+^ effector T cells, suggesting productive antigen-priming and T-cell activation. Conversely, resistant tumors demonstrated SPP1^+^ macrophage-driven immunosuppressive signaling to naïve and exhausted CD8^+^ T cells, as well as enhanced regulatory T-cell (Treg) interactions with CD8^+^ naïve cells—both features consistent with immune exclusion and checkpoint resistance.

Pathway-level analysis revealed distinct ligand–receptor usage patterns across resistant and sensitive tumors (Fig. 5e). In sensitive tumors, immune-activating modules—including Galectin, Midkine (MK), CCL and CXCL chemokines, and Annexin—were upregulated, consistent with T and NK cell recruitment and cytotoxic priming. Notably, LIGHT (TNFSF14), a T-cell co-stimulatory signal, was elevated in sensitive lesions, supporting its role in enhancing ICI efficacy. In contrast, resistant tumors exhibited increased signaling via immune-suppressive or pro-tumor axes such as PLAU, TGF-β, VEGF, SPP1, and MIF. Among these, MIF emerged as a dominant cytokine in resistant tumors, aligning with its known role in inducing M2 macrophage polarization and promoting immune evasion. Additional resistant-associated signals—including CypA, Semaphorin (SEMA3), and Visfatin—further underscored the presence of a matrix-remodeling and immunosuppressive niche.

Finally, a focused evaluation of paracrine signaling networks revealed distinct WNT and TGF-β axis utilization across response groups (Fig. 5f). Canonical TGF-β ligands and their cognate receptors (e.g. TGFB1/2/3–TGFBR1/2) were preferentially enriched in sensitive tumors, possibly reflecting feedback-driven activation in an inflamed context. Conversely, noncanonical TGF-β signals—particularly those involving TGFB2/3–ACVRL1 and related partners—were elevated in resistant tumors. Similarly, noncanonical WNT ligands (WNT5A, WNT5B, and WNT11) and their receptors (e.g. FZD4 and FZD6) were predominantly active in the resistant group, consistent with enhanced tumor–macrophage crosstalk and prior evidence linking these axes to macrophage-mediated resistance.

Together, these findings indicate that pembrolizumab sensitivity is associated with enriched immune–immune communication, immune-activating signaling pathways, and spatial/functional exclusion of immunosuppressive TAMs. In contrast, resistant metastatic sites are dominated by epithelial-centric and M2-like macrophage-driven suppressive circuits, marked by noncanonical TGF-β and WNT signaling. These intercellular signaling landscapes may underpin the differential therapeutic responsiveness observed across metastatic niches in dMMR CRC.

**Figure 6:**
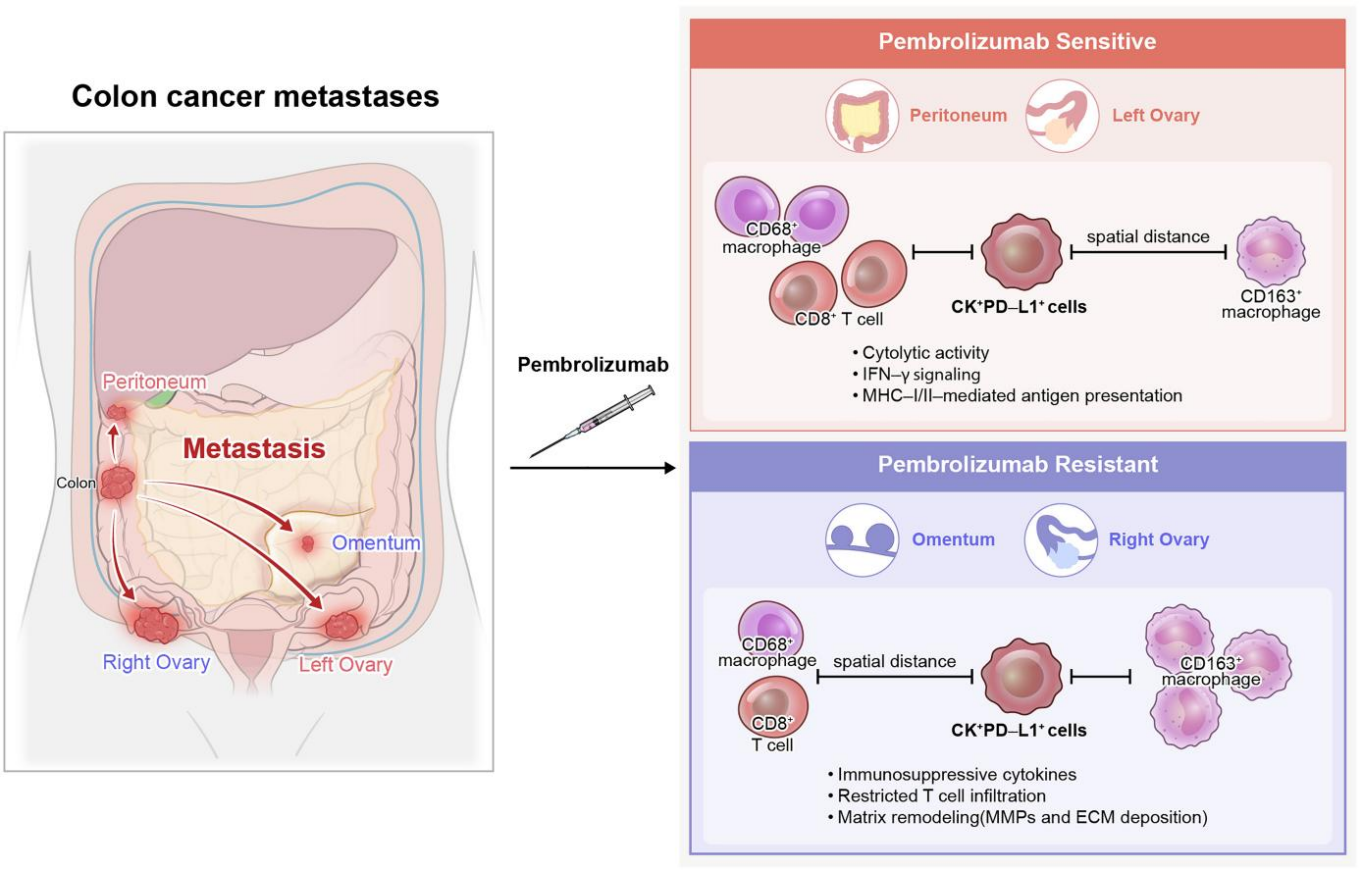
Integrated spatial–molecular model of metastatic dMMR CRC and pembrolizumab response. (a) Schematic overview of primary colon carcinoma dissemination to four metastatic niches—peritoneum, omentum, left ovary, and right ovary—prior to therapy, and the subsequent divergence into pembrolizumab-sensitive (peritoneum and left ovary) and -resistant (omentum and right ovary) phenotypes following treatment. In sensitive lesions, CK^+^PD-L1^+^ tumor cells are tightly colocalized with CD68^+^ M1-like macrophages and CD8^+^ T cells, exhibit elevated cytolytic activity, IFN-γ signaling, and MHC-I/II-mediated antigen presentation, and spatially exclude CD163^+^ M2-like macrophages. By contrast, resistant lesions are characterized by CK^+^PD-L1^+^–CD163^+^ proximity, restricted CD8^+^ T-cell infiltration, and enrichment of immunosuppressive cytokines and matrix-remodeling factors (e.g. MMPs), encapsulating site-specific ICI efficacy in dMMR CRC.

Building on these observations, Fig. 6 presents an integrated spatial–molecular model in which primary colon carcinoma seeds four metastatic niches—omentum, peritoneum, and bilateral ovaries—that bifurcate into pembrolizumab-sensitive (peritoneum and left ovary) and -resistant (omentum and right ovary) cohorts. In sensitive sites, CK^+^PD-L1^+^ tumor cells are tightly colocalized with CD68^+^ M1-like macrophages and CD8^+^ T cells, exhibit heightened cytolytic, IFN-γ, and MHC-I/II antigen-presentation programs, and spatially exclude CD163^+^ M2-like macrophages. In contrast, resistant lesions display CK^+^PD-L1^+^–CD163^+^ proximity, impaired effector cell infiltration, and enrichment of immunosuppressive cytokine networks and matrix-remodeling factors (e.g. MMPs), thereby encapsulating site-specific ICI efficacy in dMMR CRC.

## Discussion

ICIs have transformed the treatment landscape for mismatch repair-deficient (dMMR) CRC, yet durable responses remain limited to fewer than half of patients (KEYNOTE-177, response rate ∼44%). Although dMMR tumors, characterized by MSI-H and high TMB, are infiltrated by T cells [21–23], therapeutic responses—particularly in metastatic settings—remain unpredictable. To address this, we performed spatial proteomic and single-cell transcriptomic analyses of multiple metastatic lesions from a single dMMR CRC patient treated with pembrolizumab.

Despite clonal origin, the metastatic lesions diverged significantly in their immune architecture and response to therapy. Based on T-cell infiltration, PD-L1 expression, and proximity of cytotoxic cells to tumor epithelium, lesions were classified as immune-high (responsive) or immune-low (non-responsive), supporting the notion that therapeutic efficacy depends not only on mutational burden but also on regional immune contexture. This classification was supported by orthogonal quantification of immune infiltration using both EPIC deconvolution of bulk RNA-seq and mIHC-based cell density measurements, which consistently showed higher CD8^+^ T-cell and macrophage infiltration in pembrolizumab-sensitive lesions. ScRNA-seq profiling showed broadly comparable proportions of major immune lineages across metastatic sites, yet the distribution of cytotoxic subsets revealed clear differences aligned with therapeutic response. Pembrolizumab-sensitive lesions (left ovary and peritoneum) displayed higher frequencies of NK cells and CD8 effector T cells, whereas resistant lesions were relatively depleted of these cytotoxic populations. This indicates that functional immune activation—not overall lineage abundance—better captures responsiveness to PD-1 blockade. In contrast, resistant lesions were enriched for CD163-high, SPP1^+^, CXCL8^+^, and CCL8^+^ macrophage states consistent with immunosuppressive remodeling. Together, these data suggest that cytotoxic effector activity and macrophage polarization, rather than bulk immune composition, are key determinants of divergent therapeutic outcomes.

Although the patient ultimately achieved long-term disease control after pembrolizumab therapy, the heterogeneous immune states observed across metastatic sites likely reflect early lesion-specific interactions with treatment rather than determinants of eventual clinical outcome. The differential immune architectures therefore provide insight into the distinct microenvironmental trajectories that each lesion engages upon exposure to PD-1 blockade, offering a mechanistic framework for understanding why sites may initially respond or remain refractory even within the same patient [24]. Although based on a single case, the lesion- specific immune divergence we observed is broadly consistent with patterns reported in multi-site immunoprofiling across other solid tumors. This concordance suggests that such spatial heterogeneity may reflect generalizable biological principles, although confirmation in larger cohorts will be essential.

A key distinguishing factor was the polarization of TAMs. Responsive lesions (e.g. peritoneum, left ovary) were enriched in M1-like macrophages expressing IL-12, TNF-α, and cytotoxic effectors, while resistant lesions (e.g. omentum, right ovary) were dominated by M2-like macrophages, particularly SPP1^+^ and TGF-β–associated phenotypes, consistent with immunosuppressive microenvironments [25]. Single-cell analysis revealed that SPP1^+^ TAMs in resistant lesions engaged in strong immunosuppressive crosstalk with T cells, while in sensitive lesions, they were spatially and functionally excluded from immune communication. C1QB^+^ macrophages showed a similar trend. In line with these transcriptional patterns, the spatial organization of CD8^+^ T cells and CD68^+^CD11c^+^ macrophages also varied across metastatic lesions (Supplementary Figure S8). Consistent with the trends observed in the proximity analysis, pembrolizumab-responsive lesions such as the left ovary and peritoneum showed greater CD8–M1 separation compared with the omentum and colon. Notably, however, the right ovary demonstrated a disproportionately large separation (>200 µm), far exceeding all other lesions. This extreme dissociation indicates that profound spatial decoupling of effector T cells from pro-inflammatory macrophages may characterize the most treatment-refractory metastatic niche, even though CD8–M1 proximity alone does not fully account for the dichotomous response pattern.

In addition, immune-high tumors exhibited enriched immune-stimulatory pathways—including Galectin, MK, CCL/CXCL chemokines, and Annexins—that promote T-cell recruitment and activation. In contrast, resistant lesions upregulated suppressive signals such as TGF-β, VEGF, SPP1, MIF, and Visfatin, often linked to immune exclusion and matrix remodeling. Canonical TGF-β signaling was more evident in sensitive tumors, whereas noncanonical pathways (e.g. WNT5A/B, SEMA3, and ANGPTL) were enriched in resistant sites.

These findings align with the broader understanding that while dMMR CRCs are generally immunogenic, responses to ICIs remain variable. Prior studies have proposed several predictive markers, including PD-L1 expression, KRAS/BRAF mutations, Lynch syndrome status, CD8^+^ T-cell infiltration, and TMB [26–28], but none sufficiently explain lesion-specific outcomes. Our study suggests that spatial immune architecture and cellular communication patterns are critical determinants of ICI response.

A key limitation of our study is the single-patient scope, which limits generalizability. Nonetheless, the multi-site profiling provides valuable insight into intrapatient heterogeneity. Future studies should expand this approach across broader cohorts to validate the observed associations.

## Conclusion

In summary, we present a spatially resolved framework that links macrophage polarization, immune contexture, and cell–cell communication to therapeutic response in metastatic dMMR CRC. These findings support incorporating spatial immune profiling into precision immunotherapy strategies for dMMR CRC.

## Supplementary Material

ltag001_Supplementary_Data

## Data Availability

Bulk RNA-seq data and Single-cell RNA-seq data are available from the corresponding author upon reasonable request and subject to institutional and ethical approvals.
