# Supplementary material for: Comprehensive spatial and immune profiling of metastatic mismatch repair–deficient colorectal cancer reveals response to immunotherapy
Source: Immunother Adv. 2026 Jan 9;6(1):ltag001. doi: 10.1093/immadv/ltag001 (PMC12898929; doi:10.1093/immadv/ltag001)
Supplement: ltag001_Supplementary_Data [file ltag001_supplementary_data.zip › Supplement_figures_121125.pdf]

Supplementary Figures

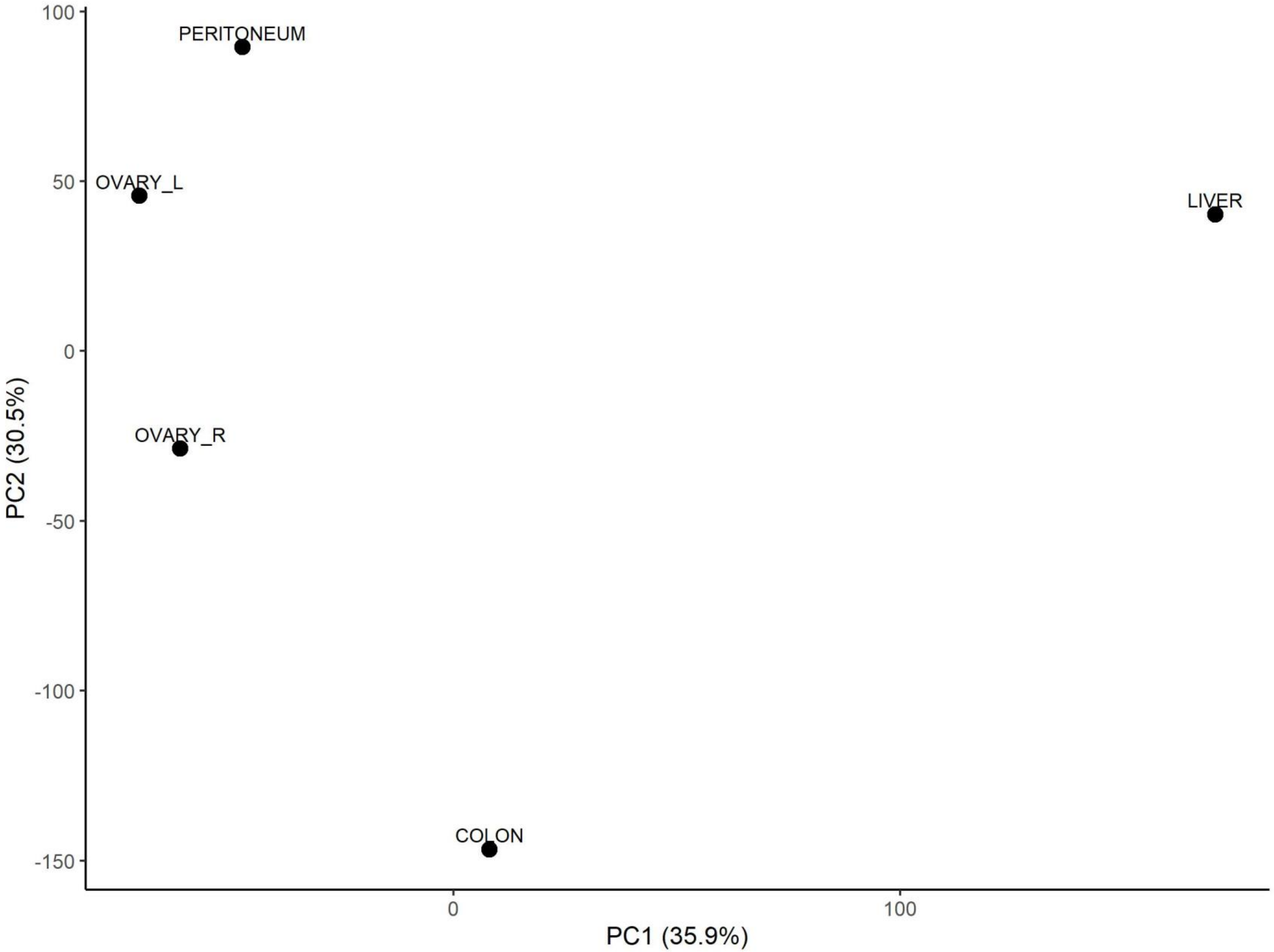

Supplementary Fig. 1. Principal component analysis of bulk RNA-seq profiles across primary and metastatic dMMR CRC lesions. PCA plot of transcriptomes derived from primary colon tumor and matched metastatic sites—including left ovary, right ovary, peritoneum, and liver. Each point represents an individual sample, colored by tissue origin, demonstrating distinct clustering by anatomical site

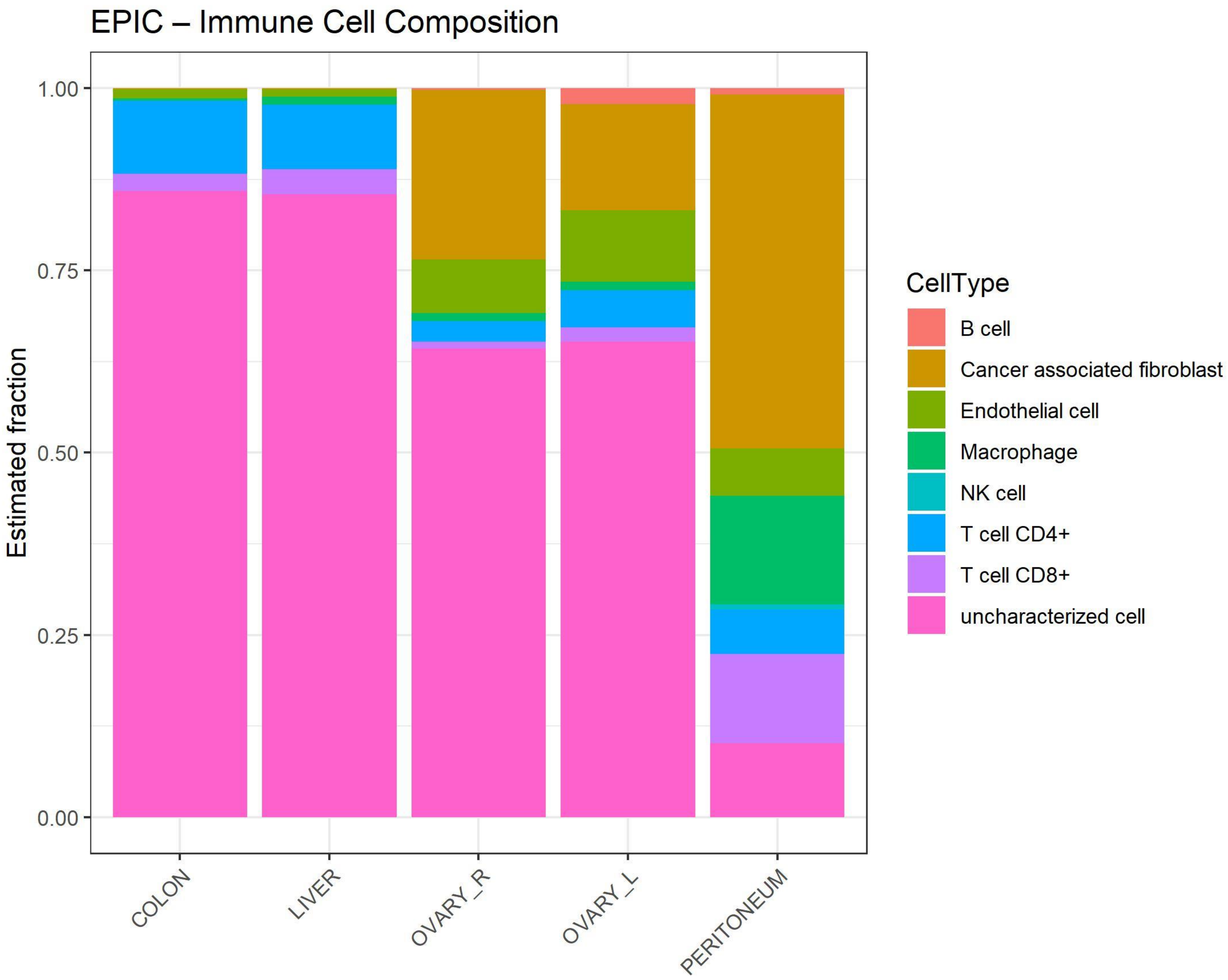

Supplementary Fig. S2. EPIC-based deconvolution of bulk RNA-seq across dMMR CRC lesions. Stacked bar plot showing inferred immune and stromal cell fractions from bulk RNA-seq using EPIC. Samples include primary colon tumor and matched liver, right ovary, left ovary, and peritoneal metastases. Pembrolizumab-responsive lesions (peritoneum and left ovary) display higher estimated T cell, macrophage, endothelial, and CAF fractions compared with resistant sites.

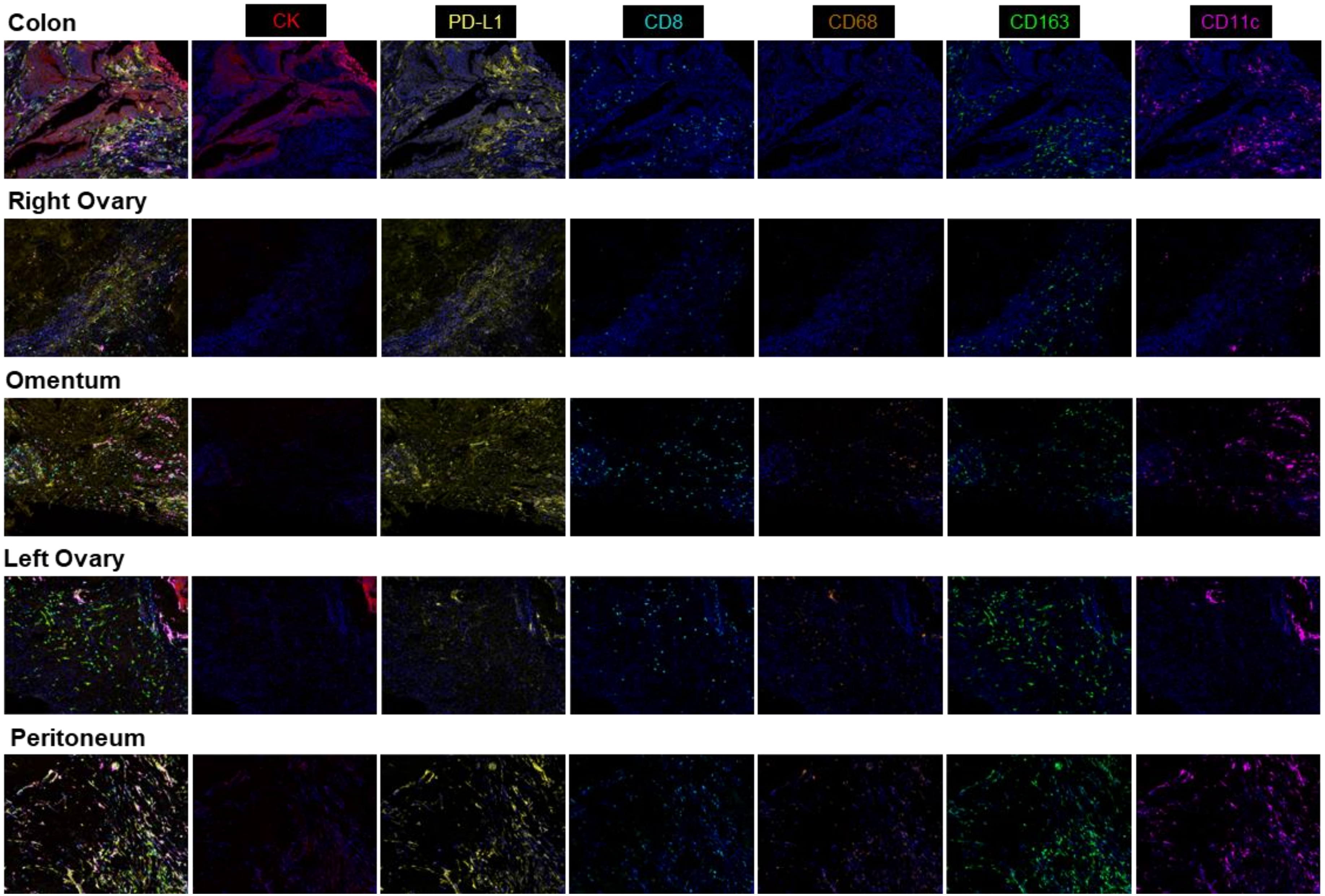

Supplementary Fig. 3-1. Multiplex immunohistochemistry profiling of epithelial and immune markers across primary and metastatic dMMR CRC lesions. Tissue sections from the primary colon tumor and matched metastases (right ovary, omentum, left ovary, and peritoneum) were stained for cytokeratin (CK), PD-L1, CD8, CD68, CD163, and CD11c. Composite overlay images (left) depict all six markers simultaneously for each site, and adjacent panels illustrate the individual spatial distribution of each marker per tissue.

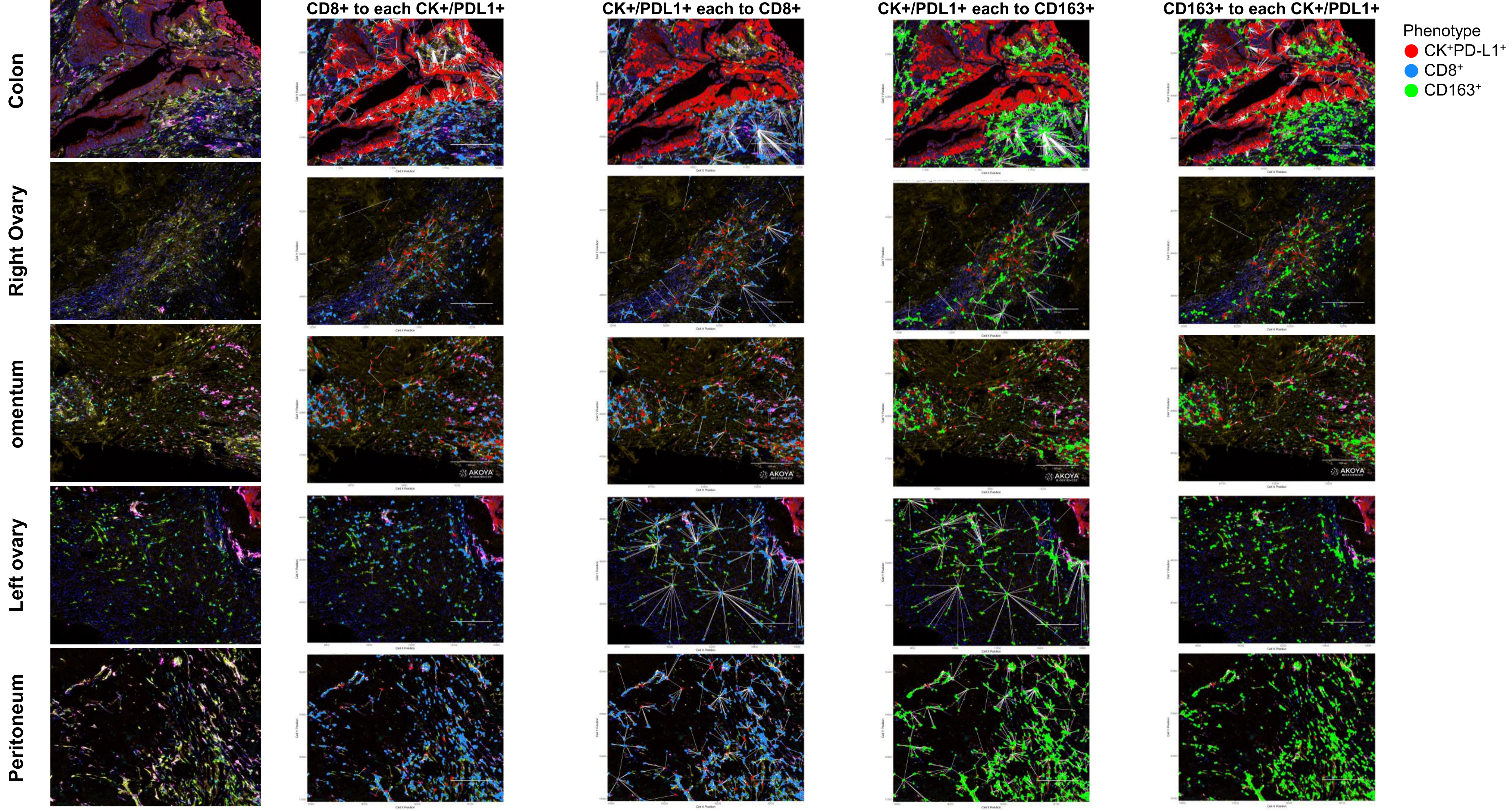

Supplementary Fig. 3-2. Representative cell segmentation, phenotype assignment, and nearest-neighbor mapping in multiplex immunohistochemistry across primary and metastatic dMMR CRC lesions.

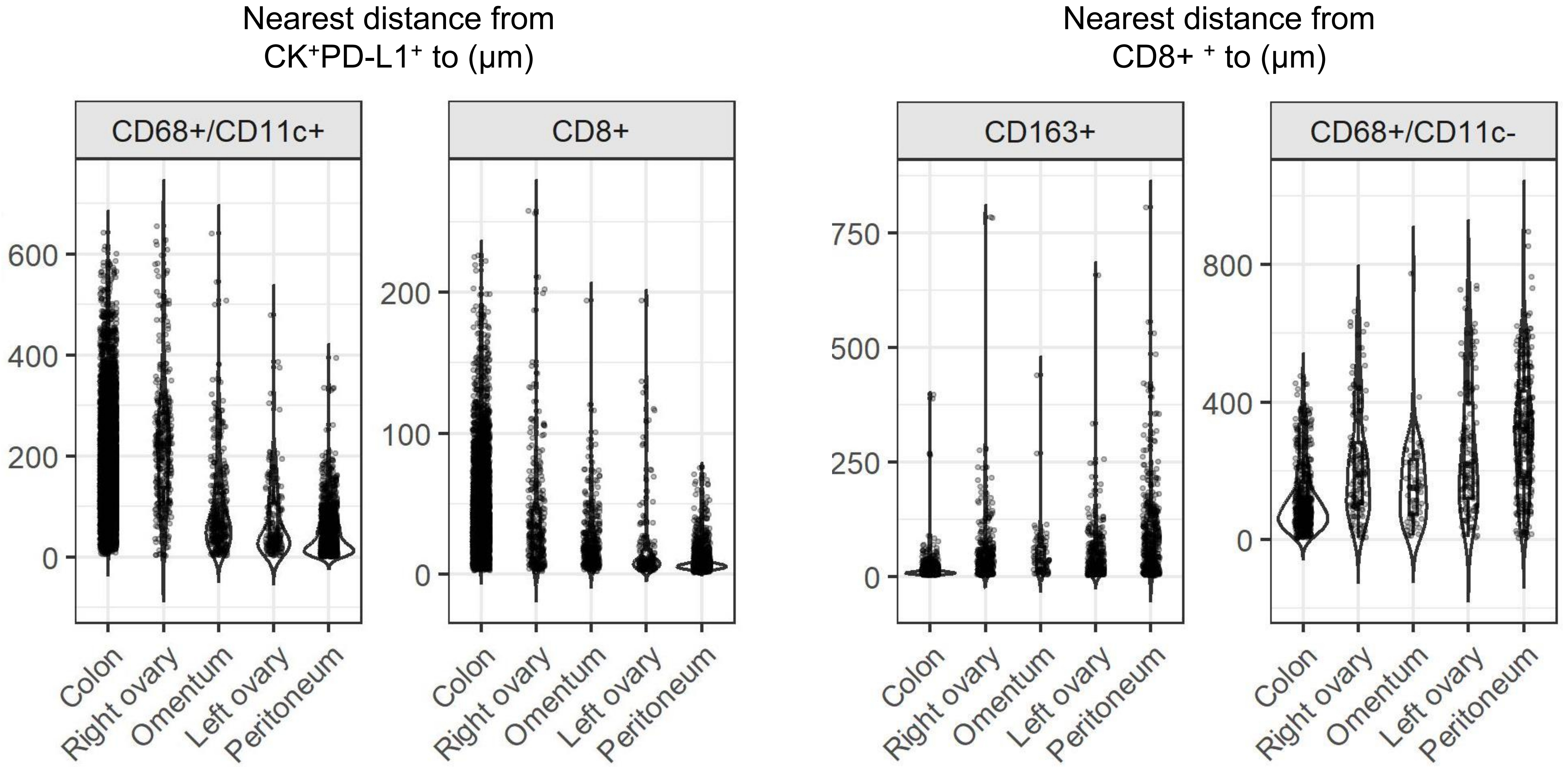

Supplementary Fig. 3-3. Nearest-neighbor distances between tumor cells and immune populations in primary and metastatic dMMR CRC lesions. Global differences across sites were statistically significant for all four metrics (one-way ANOVA, $p < 2\times10^{-16}$), with a monotonic trend consistent with pembrolizumab responsiveness confirmed by Jonckheere–Terpstra test ($p < 2\times10^{-16}$).

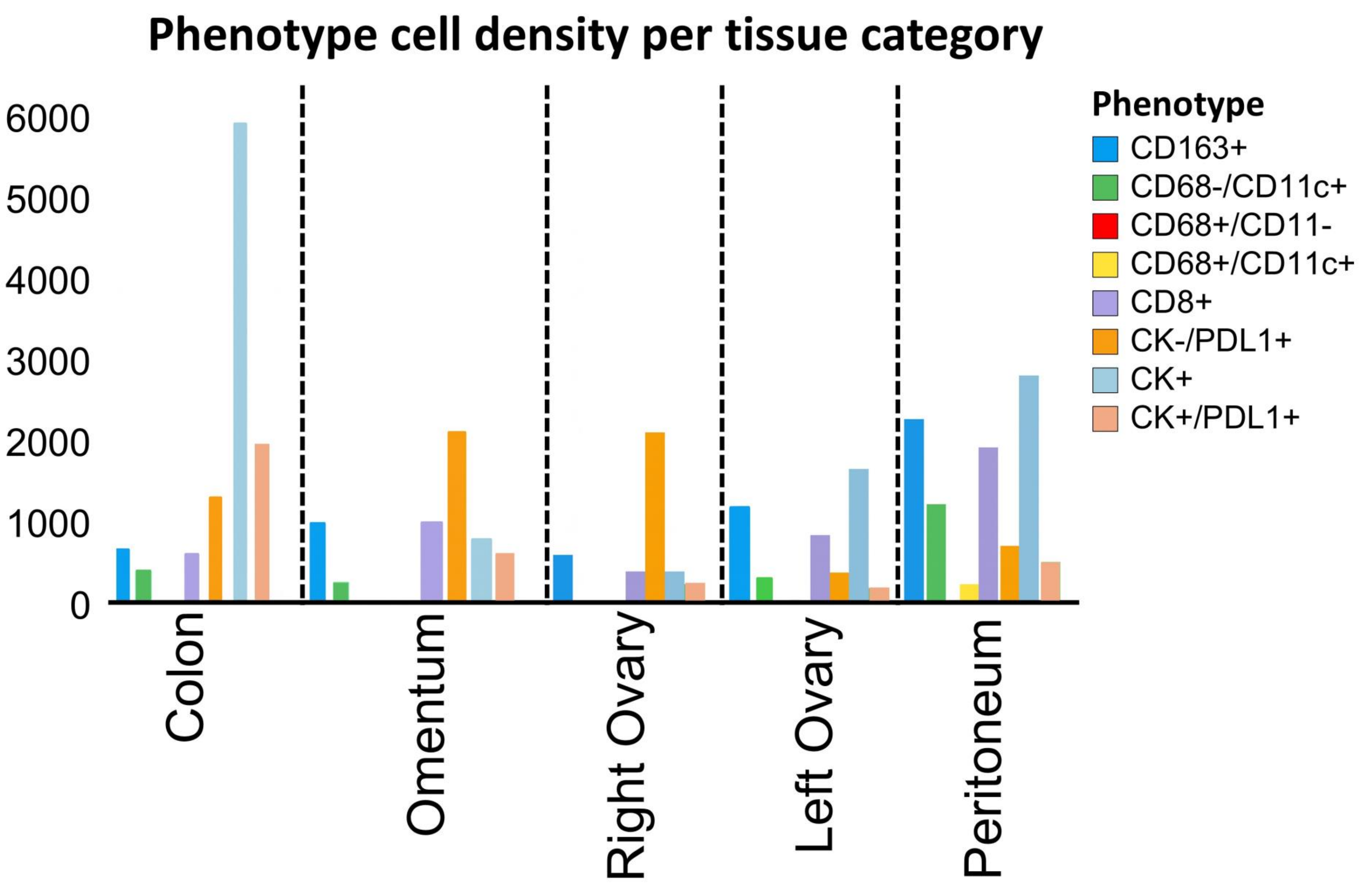

Supplementary Fig. S4. Density of mIHC-defined immune phenotypes across metastatic dMMR CRC lesions.Cell density (cells/mm²) of $CD8^{+}$ T cells, $CD68^{+}CD11c^{+}$ M1-like macrophages, $CD163^{+}$ macrophages, and CK^{-}/PD-$L1^{+}$ stromal/immune cells quantified from multiplex immunohistochemistry and normalized to tumor area. Responsive lesions (peritoneum and left ovary) show higher effector cell density compared with resistant sites.

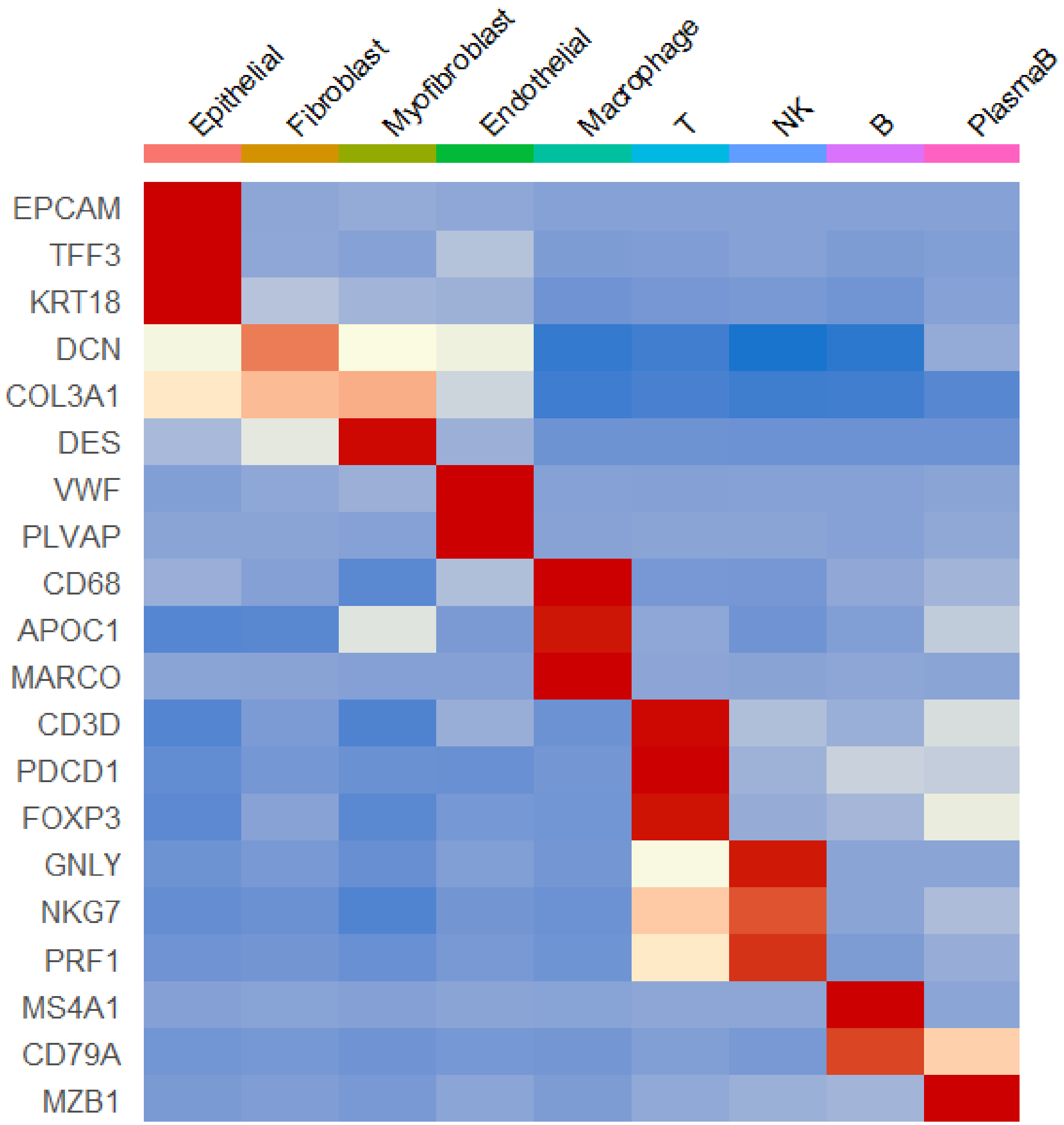

Supplementary Fig. 5. Heatmap validation of single-cell RNA-seq cell type annotations.Scaled expression (Z-score) of canonical marker genes is shown for each annotated cell cluster. Rows correspond to marker genes selected to define major populations (e.g., epithelial, CD8$^+$ T, CD4$^+$ T, NK, macrophage, dendritic, B and plasma cells), and columns represent the respective cell clusters. This heatmap confirms robust marker enrichment and accurate cell type assignment.

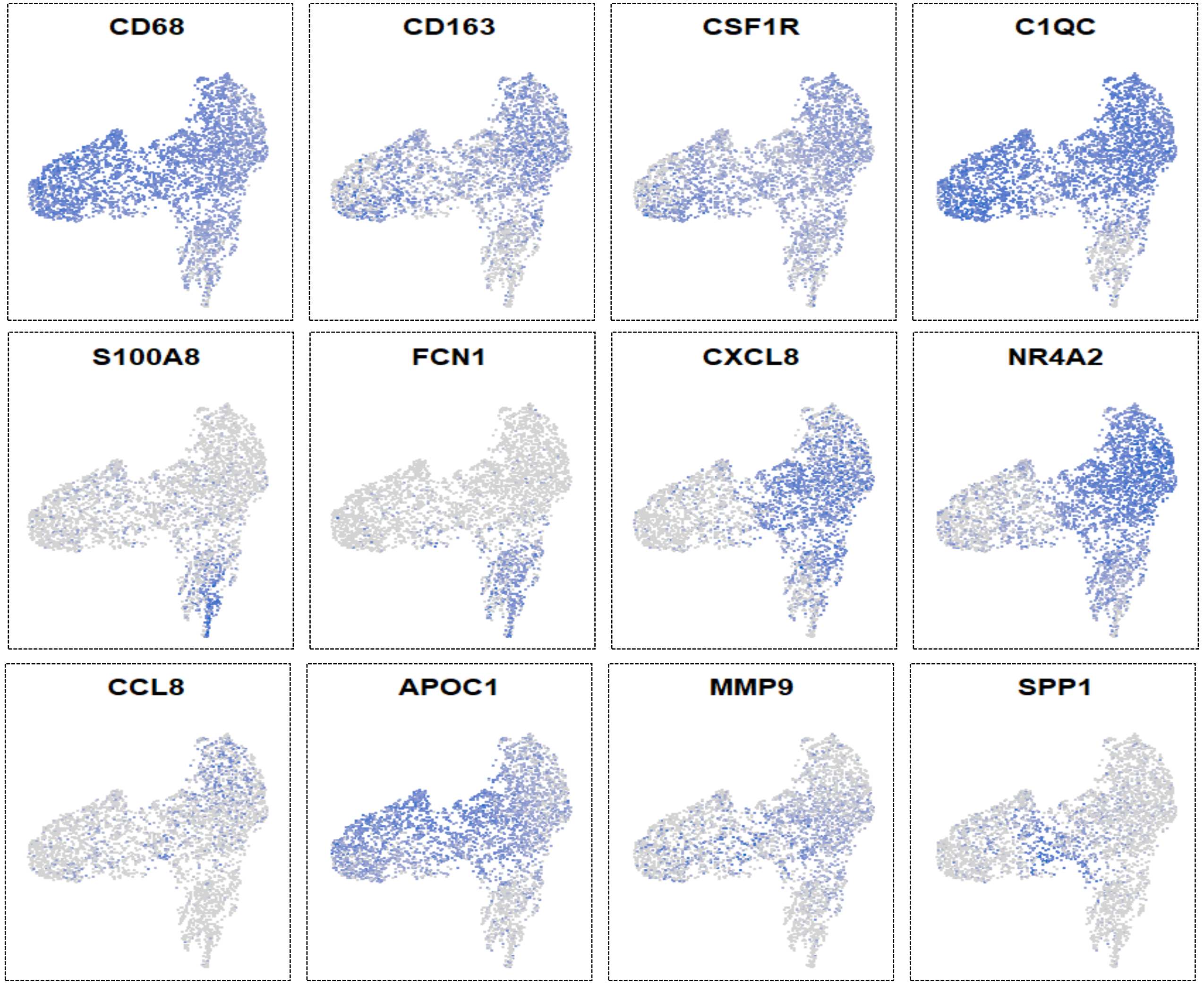

Supplementary Fig. 6. Dimensional reduction visualization of macrophage subclusters and marker-based annotation. UMAP of single-cell RNA-seq data restricted to the macrophage compartment, showing 12 distinct clusters. Each cluster is annotated by overlaid expression of its defining marker genes, confirming the identity and spatial arrangement of macrophage subtypes across metastatic dMMR CRC samples.

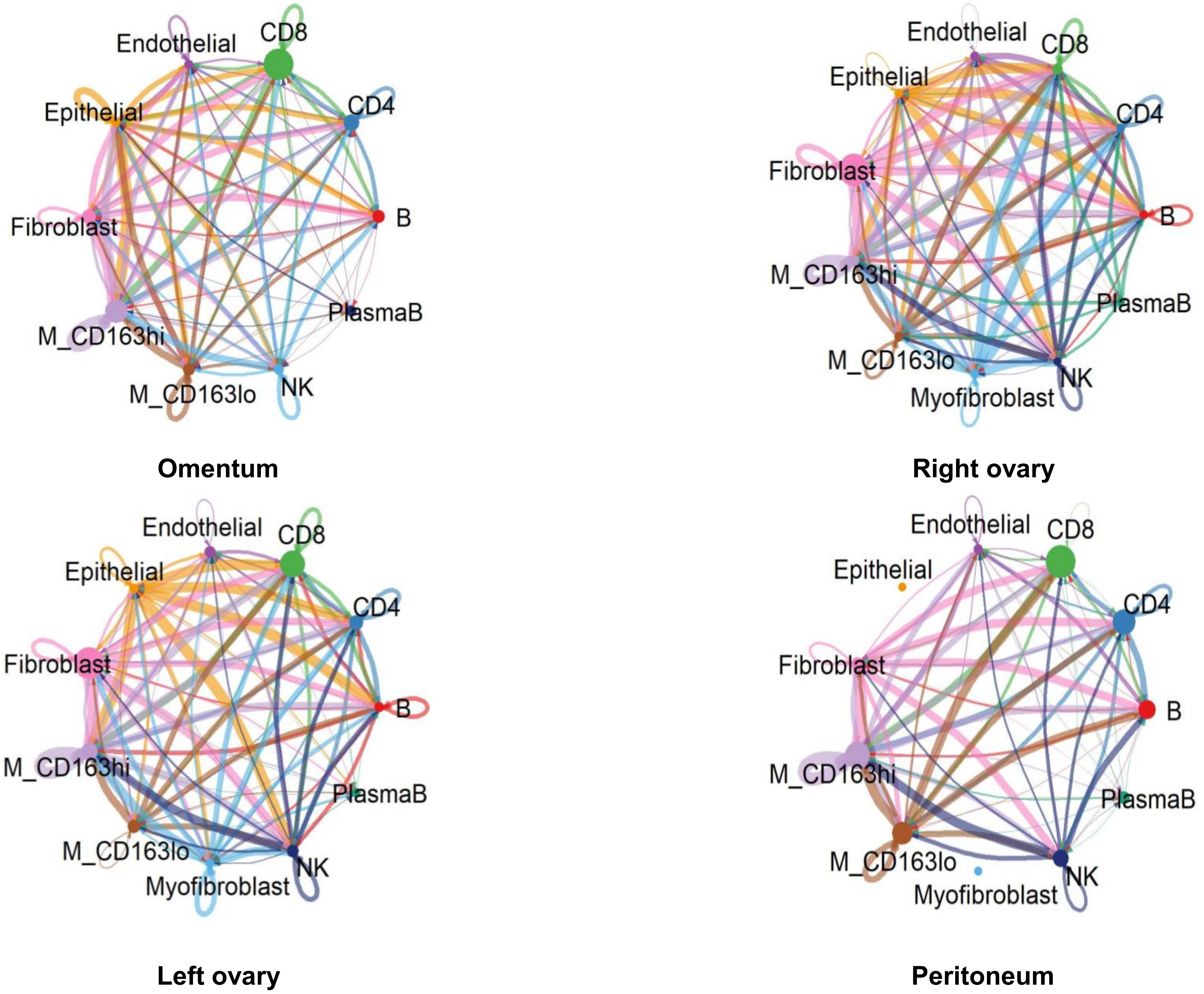

Supplementary Fig. 7. Predicted intercellular communication networks across metastatic niches in dMMR CRC. Cell–cell interaction circle plots for omentum, right ovary, left ovary, and peritoneum illustrating major cell populations and their predicted ligand–receptor signaling crosstalk; edge thickness corresponds to the strength of predicted interactions.

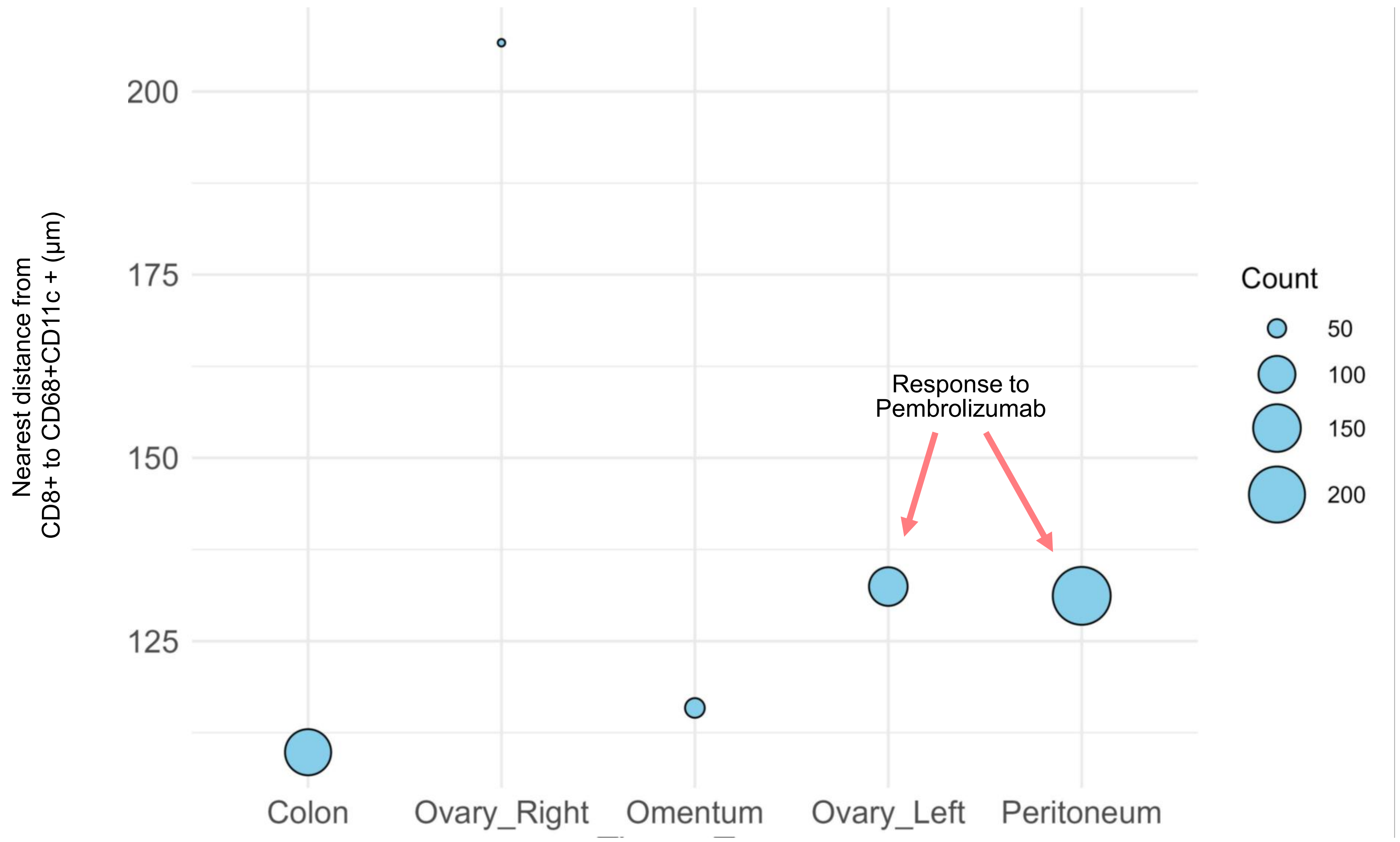

Supplementary Fig. 8. Nearest–Neighbor Distances Between CD8⁺ T Cells and CD68⁺CD11c⁺ Macrophages Across Metastatic Sites. Quantitative spatial analysis of the minimum Euclidean distance (in µm) between CD8⁺ cytotoxic T cells and CD68⁺CD11c⁺ M1-like macrophages across the metastatic lesions.
